# Transdiagnostic Psychopathology in a Help-Seeking Population of an Early Recognition Center for Mental Disorders: Protocol for an Experience Sampling Study

**DOI:** 10.2196/35206

**Published:** 2022-08-01

**Authors:** Marlene Rosen, Linda T Betz, Christian Montag, Christopher Kannen, Joseph Kambeitz

**Affiliations:** 1 Department of Psychiatry and Psychotherapy Faculty of Medicine and University Hospital of Cologne University of Cologne Cologne Germany; 2 Institute of Psychology and Education Ulm University Ulm Germany

**Keywords:** help-seeking population, phenotyping, ecological momentary assessment, symptom networks, transdiagnositc psychiatry, prevention, early intervention, psychiatry, mental health

## Abstract

**Background:**

Prevention in psychiatry provides a promising way to address the burden of mental illness. However, established approaches focus on specific diagnoses and do not address the heterogeneity and manifold potential outcomes of help-seeking populations that present at early recognition services. Conceptualizing the psychopathology manifested in help-seeking populations from a network perspective of interacting symptoms allows transdiagnostic investigations beyond binary disease categories. Furthermore, modern technologies such as smartphones facilitate the application of the Experience Sampling Method (ESM).

**Objective:**

This study is a combination of ESM with network analyses to provide valid insights beyond the established assessment instruments in a help-seeking population.

**Methods:**

We will examine 75 individuals (aged 18-40 years) of the help-seeking population of the Cologne early recognition center. For a maximally naturalistic sample, only minimal exclusion criteria will be applied. We will collect data for 14 days using a mobile app to assess 10 transdiagnostic symptoms (ie, depressive, anxious, and psychotic symptoms) as well as distress level 5 times a day. With these data, we will generate average group-level symptom networks and personalized symptom networks using a 2-step multilevel vector autoregressive model. Additionally, we will explore associations between symptom networks and sociodemographic, risk, and resilience factors, as well as psychosocial functioning.

**Results:**

The protocol was designed in February 2020 and approved by the Ethics Committee of the University Hospital Cologne in October 2020. The protocol was reviewed and funded by the Köln Fortune program in September 2020. Data collection began in November 2020 and was completed in November 2021. Of the 258 participants who were screened, 93 (36%) fulfilled the inclusion criteria and were willing to participate in the study. Of these 93 participants, 86 (92%) completed the study. The first results are expected to be published in 2022.

**Conclusions:**

This study will provide insights about the feasibility and utility of the ESM in a help-seeking population of an early recognition center. Providing the first explorative phenotyping of transdiagnostic psychopathology in this population, our study will contribute to the innovation of early recognition in psychiatry. The results will help pave the way for prevention and targeted early intervention in a broader patient group, and thus, enable greater intended effects in alleviating the burden of psychiatric disorders.

**International Registered Report Identifier (IRRID):**

DERR1-10.2196/35206

## Introduction

### Background

Prevention and early intervention in psychiatry provide promising ways to address the immense burden of mental illness [1-3]. The currently established prevention approach implemented in early recognition services focuses on risk syndromes developed for predicting specific diagnoses (eg, psychosis [4,5]). However, the majority of help-seeking patients who present at early recognition services are not covered by these specific risk syndromes, as they do not fulfill the respective criteria that indicate the increased risk that qualifies them for targeted intervention [4,6]. Thus, in a sizable proportion of this population, early recognition centers for mental disorders currently miss out on a critical potential for preventive efforts. In fact, help-seeking populations present with a mixture of various symptoms [7] such as depressive, anxious, and psychotic symptoms. Depressive and anxiety symptoms have proven to be among the main reasons why individuals seek help [8,9], whereas psychotic symptoms are of interest as they are the most burdensome for the affected individuals as well as for the health care system, despite their low prevalence [10]. These symptoms are shared across different diagnoses [11-13], as well as different disorder states such as risk-syndrome, subthreshold, and full-threshold disorders [11]. Similarly, growing evidence demonstrates that distress is a mediating and triggering factor for psychopathology at large [12-16]. Taken together, help-seeking populations are much more heterogeneous than previously assumed and may develop manifold potential outcomes [17] or show other unfavorable outcomes such as persisting deficits in psychosocial functioning [18].

Thus, there is a growing call for a broader, transdiagnostic approach for prevention in psychiatry [19-22]. Although there is important data on the psychopathology of patients presenting to early recognition centers (eg, [6,8,23,24]), their interpretation is limited by the typically purely cross-sectional, retrospective, and diagnosis-specific character of the assessments. However, symptoms fluctuate over time [25-27], and important insights are missed when neglecting this dynamic component of psychopathology in help-seeking populations. Moreover, as outlined above, conventional assessments are often considered in isolation rather than in concert, neglecting the transdiagnostic, intertwined nature of psychopathology in help-seeking populations. Collectively, these observations underline the necessity to use novel methods to enrich traditional self- and observer-based reports to understand the psychopathology in help-seeking populations.

One novel method consists of integrating 2 distinct innovative ideas that have emerged in the field of psychopathology in recent years [28]. The first idea consists of intensive longitudinal measurements of symptoms and other relevant variables via the Experience Sampling Method (ESM), which has become increasingly feasible and accepted in recent years, especially with the advance of mobile technology such as smartphones [29,30]. ESM provides valid insights into psychopathology as it occurs in daily life by assessing the targeted phenomena repeatedly during the course of the day within a specific time period. ESM increases ecological validity compared to retrospective reporting, reduces biases resulting from false memory or aggregation processes of experience over a longer time period, and allows the collection of data at the within-person level [31].

The second idea is the network approach mainly put forward by Borsboom et al [32-34] (recent overview [35]), in which psychopathology is conceived as a dynamic system of connected, interacting, and maintaining symptoms and other clinically relevant variables [32,36]. In line with a clinician’s perspective, symptoms are assumed to co-occur because of functional relations between them rather than due the common dependence on an underlying disorder entity [33,35,36]. With its inherently transdiagnostic outlook [34,37], the network approach is well suited for conceptualizing the psychopathology of help-seeking populations, where the patterns and strength of symptom expression is typically highly heterogeneous.

The integration of network analyses with ESM data enables rich insights beyond those obtained by established assessment instruments. Specifically, the intensive time-series data that result from ESM can be used to model symptom networks that offer a promising gateway into understanding the dynamics of psychopathology on the group and individual levels [38]. On the group level, dynamic symptom networks allow us to exploratively map out the potential average causal relations among individual symptoms in the same measurement window and across measurement windows. Personalized symptom networks are of special interest, as they allow the conceptualization of psychopathology as a set of person-specific dynamic processes [36,39]. By revealing the symptoms and processes most relevant to each individual, these approaches hold the potential to personalize interventions [36,40].

Due to these properties, many interesting studies have been published proving the potential of longitudinal symptom network models in advancing the psychopathological understanding of specific psychiatric conditions [41,42]. However, insights into the dynamic structure of psychopathology of a heterogeneous help-seeking population of a psychiatric early recognition center—the interactions of a broad, transdiagnostic set of symptoms, as well as the associations with risk and resilience factors and psychosocial functioning—are still lacking so far.

Thus, with this proposed study, we aim to provide the first explorative, transdiagnostic phenotyping through the combination of ESM with network analyses. This will be the one of the first studies aimed at phenotyping the transdiagnostic help-seeking population of an early recognition center for mental disorders by applying ESM.

Findings from this innovative approach integrated with those derived from established assessments represent a promising way to address a larger proportion of the help-seeking population as compared to current diagnosis-specific strategies aimed at preventing the burden of psychiatric disorders. Moreover, the results will have their core value in generating hypotheses regarding central dynamic psychopathological processes. These provide a basis for follow-up work dedicated to informing preventive interventions by testing experimentally whether the interventions on particular symptoms or processes lead to changes consistent with the estimated network model [43].

### Aim

The PhenoNetz study aims to explore the transdiagnostic phenotyping of a help-seeking population of an early recognition center for mental disorders using innovative, intensive longitudinal data collection via a smartphone app. A better understanding of the relevant psychopathology in this population is of great relevance given the lack of adequate interventions [44]. Combining ESM with network analyses allows for unique insights into the as yet underresearched early transdiagnostic psychopathological processes in the help-seeking population of an early recognition center of mental disorders, as well as to explore their association with risk, resilience, and psychosocial functioning.

## Methods

### Setting and Participants

In total, 100 participants will be recruited from the help-seeking population presenting at the early recognition center of mental disorders at the University Hospital of Cologne (Früherkennungs- und Therapiezentrum; FETZ) [45], with an expected dropout rate of 25% leading to a total of 75 participants in the final sample. Dropouts include the participants that withdraw from the study, are no longer reachable, or terminate the study without a sufficient number of ESM measurements (for details, see the data analysis section). The FETZ offers specialist diagnostics for the early recognition of mental disorders, with a focus on severe mental illness, in particular psychotic disorders. However, the first contact is independent of this focus and accessible for all people aged 18-40 years that have noticed any changes in their experience and behavior. Most patients find out about the FETZ through internet research or are referred by health care practitioners.

For a naturalistic characterization of the help-seeking population presenting at the FETZ using ESM, we will not impose specific inclusion criteria for participation in the PhenoNetz study. Similarly, to ensure the validity of the obtained data, only a small part of the help-seeking participants will be excluded based on the following criteria:

- acute suicidal thoughts
- IQ ≤ 70
- aged >40 years
- known previous illness of the central nervous system, as well as untreated, unstable somatic illnesses with known effects on the central nervous system (eg, untreated hypothyroidism)
- insufficient knowledge of the German language

### Procedure and Materials

All patients presenting at the FETZ not fulfilling any of the listed exclusion criteria will be addressed either directly at the FETZ or via telephone or email (given permission to contact was obtained by the clinical personnel at the FETZ) and informed about the background, goal, design, risks, benefits, and data security aspects of the study. Any open questions the participants might have will be answered directly by one of the primary investigators (MR and LTB). All willing participants will provide written informed consent prior to their participation in the study. All participants will be compensated with €40 (US $42.08) for their participation. Participants can withdraw from the study at any time without negative consequences.

**Figure 1:**
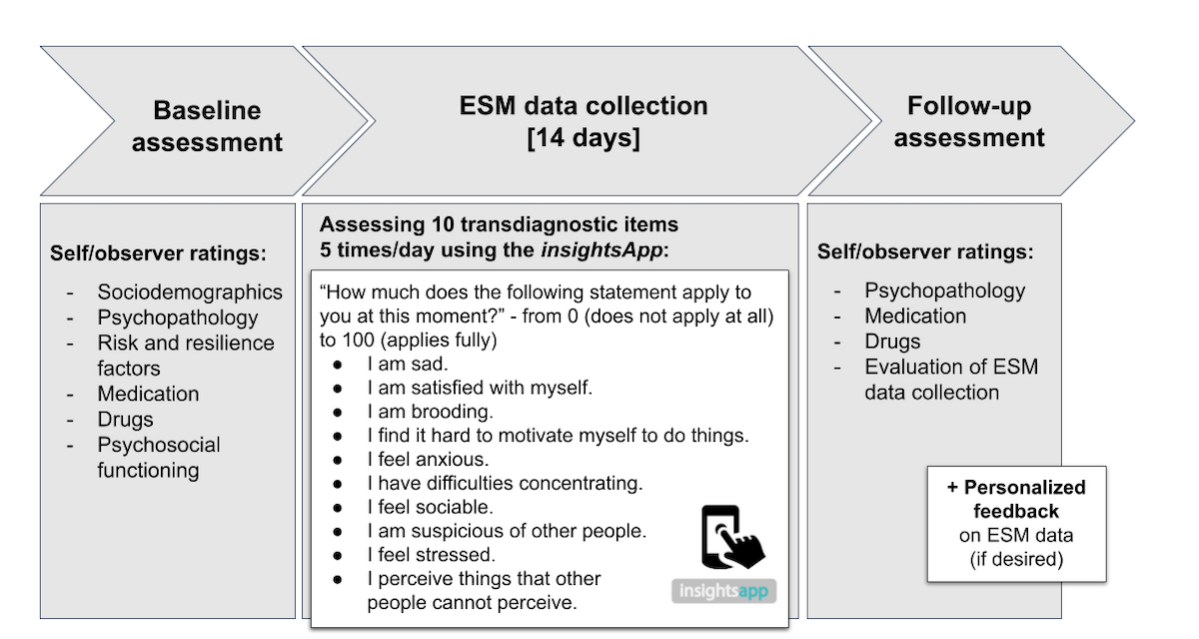
Study design of the PhenoNetz study. Participants included will undergo baseline assessment with self- and observer-ratings, followed by a 14-day ESM data collection period. In the subsequent follow-up assessment, selected self- and observer-ratings will be collected again. If desired, the participants will receive personalized feedback on their ESM data after the 2 weeks of ESM data collection, such that the feedback does not interfere with ESM data collection. ESM: Experience Sampling Method.

**Figure 2:**
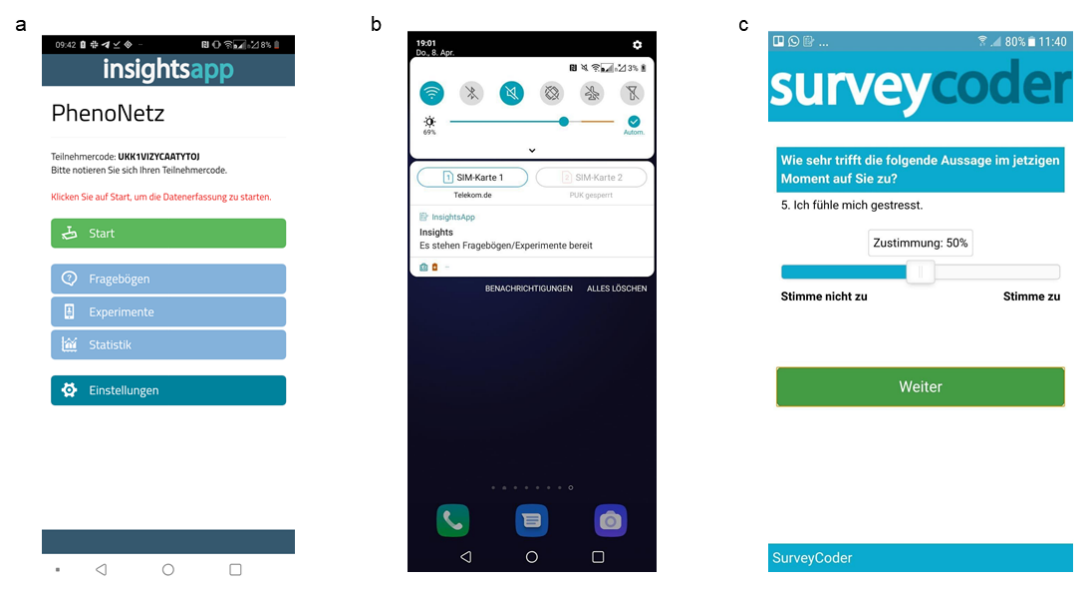
Layout of the *insightsApp*. (a) Main menu; (b) In-app reminder; (c) Visual analogue scale for answering transdiagnostic items.

Figure 1 illustrates the study design. During the baseline assessment, data on sociodemographics, medication, substance use, psychopathology including psychosocial functioning, as well as risk and resilience factors will be assessed through both observer- and self-ratings (Table 1). All data will be collected via the Research Electronic Data Capture (REDCap) software [46]. In the baseline assessment, the mobile app used for ESM data collection in the study, *insightsApp* [47] (Figure 2a), will be installed on the personal smartphones of the participants. As the *insightsApp* only runs on Android devices, participants with personal smartphones using other operating systems (eg, iOS) will be provided with a study smartphone for the study period. Participants will be encouraged to complete as many surveys as possible without substantial inconvenience or compromising their personal safety (eg, disrupting sleep or while driving). Compensation for participation will not depend on the number of completed assessments.

**Table 1:** Constructs with scales assessed at the baseline and follow-up assessments (before and after the Experience Sampling Method [ESM] period, respectively) of the PhenoNetz study.

Construct		Questionnaire		Self- vs observer -rating		Baseline assessment		Follow-up assessment
Sociodemographics		Self-designed questionnaire assessing gender, age, primary language, nationality, current living or housing conditions, highest level of education, highest vocational degree, current employment/professional activity, marital status/partnership, number of siblings, highest level of education of primary caregivers, highest vocational degree of primary caregivers		Observer-rating		✓		
**Psychopathology**								
	Diagnostic classification	Structured Clinical Interview for DSM-5 (Diagnostic and Statistical Manual of Mental Disorders, 5th Edition) [48]	Observer-rating		✓			
	Current substance use	Analogous to the Personalized Prognostic Tools for Early Psychosis Management study [49]	Observer-rating		✓		✓	
	Current medication	Analogous to the Personalized Prognostic Tools for Early Psychosis Management study [49]	Observer-rating		✓		✓	
	Depression	Beck Depression Inventory [50]	Self-rating		✓		✓	
	Anxiety	State and Trait Anxiety Inventory [51]	Self-rating		✓		✓	
	Social phobia	Social Phobia Inventory [52]	Self-rating		✓		✓	
	Psychotic symptoms	Community Assessment of Psychic Experience [53]	Self-rating		✓		✓	
	Quality of life	World Health Organization Quality of Life Questionnaire [54]	Self-rating		✓		✓	
**Risk and resilience**								
	Childhood trauma	Childhood Trauma Questionnaire [55]	Self-rating		✓			
	Bullying	Bullying Scale [56]	Self-rating		✓			
	Resilience	Resilience Scale for Adults [57]	Self-rating		✓			
	Coping	Coping Inventory for Stressful Situations [58]	Self-rating		✓			
	Personality	NEO-Five Factor Inventory [59]	Self-rating		✓			
	Attachment	Attachment Style Questionnaire [60]	Self-rating		✓			
	Expressed emotion	Level of Expressed Emotion Scale [61]	Self-rating		✓			
	Social support	Multidimensional Scale of Perceived Social Support [62]	Self-rating		✓			
	Introspection	Self-Reflection and Insight Scale [63,64]	Self-rating		✓		✓	
	Self-efficacy	Self-Efficacy Scale [65]	Self-rating		✓			
Psychosocial functioning		Global Functioning Social and Role Scales [66]	Observer-rating		✓			
Experience with ESM period		Adapted from Frumkin et al [67]	Self-rating				✓	

Using ESM, potentially relevant transdiagnostic (subthreshold) symptoms such as sadness, anxiety, psychotic experiences, and stress will be recorded (Textbox 1). The items are based on previous studies and questionnaires, given the lack of standardized ESM assessment in clinical populations [43,68]. In-app reminders will be sent out 5 times a day at fixed time points: 9:30 AM, 12:30 PM, 3:30 PM, 6:30 PM, and 9:30 PM, for a duration of 14 days (Figure 2b). Fixed sampling schemes are common in network applications to ESM data [43,67,69-71], given that they lead to equidistant measurements, an important assumption of 2-step multilevel vector autoregressive (mlVAR) modeling [38]. In psychiatric populations, fixed sampling schemes have also been associated with increased compliance [72]. In each survey, participants will be asked how much they endorse a certain feeling or behavior at the time of filling out the survey: “Wie sehr trifft die folgende Aussage im jetzigen Moment auf Sie zu?” (How much does the following statement apply to you at this moment?). Responses will be given on a visual analogue scale (as a percentage) from 0=“trifft überhaupt nicht zu” (does not apply at all) to 100=“trifft voll und ganz zu” (applies fully), with a slider that can be moved in 1-unit increments (Figure 2c). Participants will be asked to fill in the items as soon as possible after receiving the in-app reminder, but no later than 60 minutes afterwards, following prior research (eg, [43,73,74]). Filling in the items takes about 1-1.5 minutes in total. Similar ESM protocols were deemed acceptable for clinical populations in prior studies [31,67,75]. The *insightsApp* will be used only for the regular, active collection of transdiagnostic symptoms by means of the described self-report questions. No personal information (such as name and phone number, etc.) or passive data are accessed, stored, or transferred by the *insightsApp*. To maximize the number of completed surveys for each participant, the participants will be contacted at least once during the assessment period to assess instruction adherence, identify any concerns associated with the method, and help the participants with any problems in completing the ESM questionnaire.

**Experience Sampling Method (ESM) items assessed in the PhenoNetz study (along with the English translation).**

1. Ich bin traurig (I am sad).
2. Ich nehme Dinge wahr, die andere Menschen nicht wahrnehmen können (I perceive things that other people cannot perceive).
3. Ich habe Schwierigkeiten, mich zu konzentrieren (I have difficulties concentrating).
4. Ich bin kontaktfreudig (I feel sociable).
5. Ich fühle mich gestresst (I feel stressed).
6. Ich bin zufrieden mit mir (I am satisfied with myself).
7. Ich fühle mich ängstlich (I feel anxious).
8. Es fällt mir schwer, mich zu Dingen zu motivieren (I find it hard to motivate myself to do things).
9. Ich bin misstrauisch gegenüber anderen Menschen (I am suspicious of other people).
10. Ich grüble (I am brooding).

In the follow-up assessment conducted after the 14 days of ESM data collection, information on psychopathology, medication, and substance use will be assessed again, referring to the 14 days during which ESM data were collected (Table 1). In addition, experiences and strain associated with the ESM data collection will be assessed via a questionnaire translated and adjusted from a previous study conducted in clinical participants [67] (Table S1 in Multimedia Appendix 1). If desired, participants will be provided with a personalized feedback report on their ESM data.

### Data Security

Using a smartphone app installed on the personal smartphone of the participants for data assessment requires particular attention to data security (a broader discussion on ethical concerns regarding digital phenotyping procedures in the psychological and psychiatric sciences have been previously described [76,77]). Therefore, subjects must provide additional consent to allow data to be collected within the app and grant the necessary permissions to the app on the smartphones (such as being notified by the app about available surveys). The ESM data collected by the *insightsApp* are pseudonymized (16-digit alphanumeric codes) and sent directly to a server hosted and maintained by a professional web hosting service after each survey. Answers to the surveys are only stored temporarily locally on the smartphones and deleted once they are transmitted to the server. To secure the data transfer from the smartphone to the server, the connection between the *insightsApp* and the backend software on the server is encrypted by the use of a Secure Sockets Layer certificate.

### Safety

Given that this study is observational, there are no direct risks associated with participation. Previous studies have demonstrated good acceptance of ESM protocols similar to the one implemented in this study. Even if participants become more aware of their symptoms due to high-frequency data collection, this does not have a negative effect in terms of worsening symptoms [31,67,78]. Participants can terminate the ESM data collection at any time without giving reasons. Participants who are acutely suicidal or a danger to others will immediately be presented to the service physician for further assessment. Should this become apparent in a telephone call, participants will be reported to the responsible social psychiatric service.

### Data Analytic Plan

All statistical analyses will be conducted in the R statistical software (R Foundation for Statistical Computing) [79]. Descriptive analysis of the sample will include mean, SD, median, and IQR as appropriate. The participants included in the analysis will be compared to those that dropped out of the study or were excluded due to too few available measurements (see sample size and the required number of ESM observations) via appropriate classes of permutation tests [80]. Changes in measures that were assessed twice, pre- and post-ESM (see Table 1), will be compared via linear mixed modeling. Prior to the analyses of ESM data, we will detrend the ESM data by fitting fixed-effects linear regression models to each ESM item, regressing out a linear trend on time (ie, general increases/decreases in items over time) and mean-center ESM items per person. We will then generate group-level and personalized networks via a 2-step mlVAR modeling approach as described in detail below. These analyses will allow us to examine symptom dynamics within multiple individuals (n>1; fixed effects) and for individual participants (n=1; random effects). Originally, we planned to estimate and analyze “truly” personalized networks solely based on data from individual participants (such as those that could be derived via a graphical vector autoregressive model [28]). However, results from a simulation study [81], published as a preprint 1 month after our study commenced, suggest that our sampling scheme potentially lacks the power to detect a nonnegligible proportion of true edges in truly personalized networks, which is why we decided to refrain from this analytic approach.

### 2-Step mlVAR Model

We will use a mlVAR model, as implemented in the R package “mlVAR.” In the mlVAR model, the average dynamical relationships on the group level are modeled as fixed effects, whereas regression coefficients are allowed to vary between patients as random effects.

First, we will estimate 3 group-level network structures including the 10 assessed symptoms, reflecting the average process of all participants (fixed effects): between-subject (an undirected partial correlation network between the means of participant’s scores, capturing, in general, whether participants high on a given node are also high on other nodes during the 2-week course of the study), contemporaneous (an undirected partial correlation network showing how symptoms relate to each other in the same window of measurement, controlling for temporal relationships), and temporal (a directed network displaying symptoms predicting each other across an approximately 3-hour lag, while controlling for all other experiences in the model at the prior measurement). Centrality will be assessed using strength centrality (indicating the summed absolute edge strengths connected to a specific node) in the contemporaneous network, and in-strength (indicating the summed absolute strengths of all incoming edges) and out-strength (indicating the summed absolute strengths of all outgoing edges) in the temporal network will be assessed using the R package “qgraph” [82].

Second, we will generate 2 types of personalized networks for each participant based on estimated random effects of the mlVAR model: a contemporaneous network and a temporal network. These personalized networks are not truly idiographic, in the sense that they borrow information from other subjects [38,41]. However, in doing so, the mlVAR model can perform well in estimating personalized networks even if the number of ESM observations is comparatively low for a particular participant. Given that the mlVAR model does not perform participant-specific model selection, all estimated personalized networks will contain all edges [38].

We will use orthogonal estimation for contemporaneous and temporal effects. For the contemporaneous and temporal group-level networks, we will use the conservative “AND-rule” approach in retaining and plotting significant edges. A detailed description of methodological details has been described previously [38,41].

Specifically, we have planned the following analyses:

1. We will compute group longitudinal networks (between-person, contemporaneous, and temporal [28]) as described above.
2. We will identify symptom centrality and unique partial correlations among symptoms in the contemporaneous and temporal group-level networks. We hypothesize that on the group level, feeling stressed will be the most central symptom in the contemporaneous network and predict most other experiences in the temporal network, given that stress experience is frequently discussed as a transdiagnostic factor in psychopathological experiences [13-16]. For the temporal network, we have no a priori hypothesis with regard to the most central item.
3. We will evaluate the degree of association between risk factors (eg, childhood trauma) and network connectivity, assessed by the global strength of personalized networks (temporal and contemporaneous) in a linear modeling approach. Based on prior research and theoretical considerations [33,83,84], we hypothesize that risk factors will be associated with increased network connectivity. Similarly, we hypothesize that poorer psychosocial functioning will be associated with increased network connectivity.
4. We will explore how the strength of specific symptom-symptom connections in individual contemporaneous and temporal networks relates to the degree of presence of specific risk and resilience factors.

### Sample Size and the Required Number of ESM Observations

Formal power analyses have not yet been worked out for group-level network models based on intensive longitudinal data. Power at the intraindividual level is a function of within-person variability; there should be sufficient variability such that the intraclass coefficient is not too close to 1, which should usually be the case when having a large number of assessments per person as in our study [85,86]. The performance of network estimation methods also depends on the unknown true network structure—the network equivalent of a true effect size in power analysis [41]. Supplementary materials from Epskamp et al [38] report simulation results for mlVAR models, showing that mlVAR models are excellent in recovering the fixed effect structures with a small amount of data, starting at 50 participants. With our targeted sample size of 75, which represents a realistic recruitment goal in the population of interest, we will surpass this threshold, leading to an adequately powered analysis for the estimation of a mlVAR model. Due to the methodological novelty of symptom networks based on intensive time-series data, there exist no guidelines on the number of ESM observations required [41]. More observations collected over a longer period of time improves the stability and validity of the results; however, this has to be balanced against the feasibility of the integration of the study into the daily lives of the participants. With 75 targeted observations collected over 14 days, our study is similar to the study designs of previous ESM projects conducted in psychiatric populations [67,70,73-75,87,88]. Following recommended guidelines [89] and prior studies [38,71], participants with fewer than one-third of the possible ESM observations (ie, 23) will be excluded from the network estimation. 

### Ethics Approval

Ethics approval was granted by the Ethics Committee of the University Hospital Cologne in October 2020 (reference number 20-1092).

## Results

Study recruitment started on November 11, 2020, and was completed on November 10, 2021. Of the 258 participants who were screened, 93 (36%) fulfilled the inclusion criteria and were willing to participate in the study. Of these 93 participants, 86 (92%) completed the study. As of May 2022, data analysis is ongoing. The first results are expected to be published in 2022.

## Discussion

### Expected Findings

This study aims to extract an explorative phenotyping of the heterogenous help-seeking population of a psychiatric early recognition center. Applying ESM, we will attempt to depict transdiagnostic symptom networks and explore their association with protective and risk factors, as well as psychosocial functioning.

The diverse and transdiagnostic character of help-seeking populations [7] limits the potential of current, narrow concepts of prevention in psychiatry [17,19]. Our exploratory study might provide a first glimpse at the dynamics between transdiagnostic symptoms as well as the associations with outcome and preceding conditions independent of diagnostic categories. Such new insights might be more valuable for alternative preventive approaches targeting a broader patient group than currently established approaches [90]. The central transdiagnostic items and processes we will identify might represent anchor points for interventions [91], which might deviate from diagnosis-specific manuals only focusing on symptoms and processes covered by diagnostic criteria [92]. Furthermore, insights into potential etiological processes, identified by the association with risk and resilience factors as well as psychosocial functioning, might inform prevention strategies [44,92]. Dynamic models based on ESM data being more in line with the true nature of psychopathology instead of static models [25,27,92] might be more effective in the prediction of outcome. In particular, transdiagnostic symptomatology was not often depicted by ESM studies so far [72]. Hypotheses based on the findings of our explorative study might guide future research.

### Strengths and Limitations

The atheoretical approach of our study facilitates truly innovative insights not biased or limited by established theories and structures. In addition, the choice for a naturalistic sample with only a limited set of inclusion and exclusion criteria is valuable for external validity.

However, there are several limitations in the design of our study that need to be considered. First, we acknowledge that the use of study smartphones may result in the underrepresentation of iPhone users in our study, as well as less valid data collection than if the participants can use their own smartphones.

The biggest challenge during the conceptualization of the study was the lack of officially validated ESM items. In general, as gold standards for the novel methodology of ESM are missing, researchers construct their own ESM items or refer to items used in previous studies [43,68,93]. Furthermore, most ESM studies focus on specific diagnoses (eg, major depressive disorder [72]). In a *transdiagnostic* ESM design, by contrast, it is difficult to cover the entire diversity of different disorders due to the further limited number of items per diagnosis-specific phenomenology. These difficulties underscore a recent call for valid and reliable scales suitable for investigating the short-term dynamics of emotions and state mental health problems [43].

### Future Directions

ESM represents only one of the various powerful elements (eg, digital phenotyping [94,95]) used to gain insights into relevant variables collected in everyday life to improve prevention and targeted early intervention. Studying the digital footprints left by the human-smartphone interaction (eg, log-in frequency, the use of different apps, and calling behavior) can provide additional important insights into the psychopathological states in help-seeking individuals [96]. Exploring the potential of ESM as a self-monitoring intervention in help-seeking populations (similar to approaches in depressive disorder [26]) is another exciting avenue for future research.

### Conclusion

In clinical science, intensive longitudinal assessments of symptoms in daily life are deservedly receiving more and more attention [36,40,41] that might result in enhanced patient benefit. By applying ESM and network analyses, our study intends to contribute a milestone toward innovation in understanding help-seeking populations in psychiatry, helping a greater proportion of this heterogeneous and crucial target group [40]. Subsequent impacts on early states and the progress of mental disorders might reduce the associated personal, familial, societal, clinical, and economic burden more effectively.

## Appendix

Multimedia Appendix 1 Questionnaire to assess experiences and strain associated with the ESM data collection translated and adjusted from a previous study conducted in clinical participants. ESM: Experience Sampling Method.

## Abbreviations

ESM: Experience Sampling Method
FETZ: Früherkennungs- und Therapiezentrum (the early recognition center of mental disorders at the University Hospital of Cologne)
mlVAR: multilevel vector autoregressive
