# Supplementary material for: Transdiagnostic Psychopathology in a Help-Seeking Population of an Early Recognition Center for Mental Disorders: Protocol for an Experience Sampling Study
Source: JMIR Res Protoc. 2022 Aug 1;11(8):e35206. doi: 10.2196/35206 (PMC9379784; doi:10.2196/35206)
Supplement: Multimedia Appendix 1 [file resprot_v11i8e35206_app1.pdf]

Supplementary material for
"Transdiagnostic psychopathology in a help-seeking population of an early recognition center: protocol for an experience sampling study"

Rosen, Betz et al.

Table S1. Questionnaire to assess experiences and strain associated with the ESM data collection translated and adjusted from a previous study conducted in clinical participants [1].

	Stimme überhaupt nicht zu	Stimme nicht zu	Weder noch	Stimme zu	Stimme stark zu
Es war mir lästig, jeden Tag fünf mal zehn Fragen zu beantworten.					
Ich war mir meiner Stimmung/ meiner Symptome bewusst.					
Das Beantworten der Fragen in der App hat mir meine Stimmung/ meine Symptome bewusster gemacht.					
Ich habe mich schlechter gefühlt, wenn ich mir meiner Stimmung/ meiner Symptome bewusst war.					
Ich habe mich besser gefühlt, wenn ich mir meiner Stimmung/ meiner Symptome bewusst war.					

Reference

1. Frumkin M, Piccirillo M, Beck ED, Grossman J, Rodebaugh T. Feasibility and utility of idiographic models in the clinic: A pilot study. Psychother Res. 2020. doi:10.1080/10503307.2020.1805133
